# Exploration of user needs and design requirements of a digital stress management intervention for software employees in Sri Lanka: a qualitative study

**DOI:** 10.1186/s12889-023-15480-7

**Published:** 2023-03-27

**Authors:** Manoja Weerasekara, Åsa B. Smedberg

**Affiliations:** 1 Department of Computer and Systems Sciences (DSV), Stockholm University, Stockholm, Sweden; 2 Department of Information and Systems Sciences, NSBM Green University, Homagama, Sri Lanka

**Keywords:** Occupational stress, ICT, Design, User needs, System feature, Focus group

## Abstract

**Background:**

Digital stress management interventions are considered promising additions to the spectrum of the programs companies use to support the well-being of their employees. However, a series of constraints are identified that hinder the potential benefits of such interventions. These constraints include a lack of user engagement and personalisation, poor adherence and high attrition. Understanding the specific user needs and requirements is essential to increase the likelihood of success in implementing ICT (Information and Communication Technology)-supported stress management interventions. Thus, following the findings from a previous quantitative study, the proposed study aimed to further explore the user needs and requirements for designing digital stress management interventions for software employees in Sri Lanka.

**Methods:**

The study used a qualitative approach based on three focus groups with 22 software employees in Sri Lanka. The focus group discussions were conducted online and recorded digitally. Inductive thematic analysis was used to analyse the collected data. The consolidated criteria for reporting qualitative studies (COREQ)-32 items were followed for reporting this study.

**Results:**

The analysis revealed three major themes: self-help in a personal space, social support in a collaborative space, and general design considerations for achieving success. The first theme revealed the users’ preference for having a personal space where they could engage in individual activities without support from an external party. The second theme elaborated on the importance of adding a collaborative platform to seek help from peers and professionals. The final theme explored the user-desired design features that could increase user engagement and adherence.

**Conclusions:**

This study used a qualitative approach to further explore the findings of a previous quantitative study. The focus group discussions confirmed the results of the previous study and provided the opportunity to better understand user needs and yield new insights. Such insights revealed user preference for embedding personal and collaborative platforms in a single intervention, embedding gamified elements, the provision of passive content generation via sensory systems, and the need for personalisation. These empirical findings will feed into the design of ICT-supported interventions for occupational stress management among Sri Lankan software employees.

**Supplementary Information:**

The online version contains supplementary material available at 10.1186/s12889-023-15480-7.

## Introduction

A recent survey [1] published by Headspace, covering over 5,000 employees from the US, UK, Australia, Germany and France, noted that the number of cases reported from individuals and employers regarding work-stress conditions has increased and highlighted this as a warning sign regarding an individual’s well-being and the company’s bottom line. Moreover, ReMark’s eighth annual Global Consumer Study (GCS) [2] estimated that 792 million individuals have lived with a mental health disorder—more than one in ten globally—for the past two years. The same report indicated that top concerns are associated with work issues, followed by financial, family, or relationship problems. Besides the impact on workers’ well-being, work-related stress can increase absenteeism and presenteeism, reduce commitment and motivation, higher staff turnover rate and increase intention to quit [3]. Though all occupations are subjected to stress, the software industry is considered more vulnerable to stressful conditions. Moreover, the software industry is regarded as a capital-intensive human industry; a high-stress level would negatively impact employees’ creativity and innovation, causing a threat to product quality. Thus, organisations and individuals must make sufficient effort to identify the best potential solution.

Recent statistics at the Mental Health Unit of Sri Lanka’s Health Ministry mirror the global stress conditions; the statistics show that one in five public sector employees suffers from stress, which has become a real issue that needs to be addressed in the workplace [4]. Though stress-related problems at the workplace remain a serious challenge within society, the general treatment uptake is considerably low [5]. This situation may have been caused by the stigma related to mental health, lack of awareness, insufficient resources, and unaffordability of consultations and treatment [6]. Due to the country’s scarcity of mental healthcare human resources, there has been particular interest in introducing innovations to deal with the situation [7]. However, a recent report compiled on the mental health campaign and advocacy in Sri Lanka highlighted that regardless of the country’s high digital literacy level, the involvement of technology in mental health aspects is low [5].

The literature and contemporary studies highlight that the growing number of digital interventions offers promising ways to deliver stress management interventions conveniently and efficiently [8–11]. Research emphasises the role digital interventions can play in providing support and care conveniently to larger audiences regardless of their geographical location [12]. Digital health interventions may vary based on focusing on a particular facet or facet of mental health. They also support multiple engagements and communication methods and offer the possibility of rendering consistent and efficient interventions at a lower cost [13, 14]. Despite the advantages embedded in digital health interventions, there are challenges attached to digital mental health interventions. These include low adherence, lack of engagement, high attrition, and lack of personalisation which hinder the benefits of such interventions [15]. Low adherence, also called non-usage attrition, occurs when the study participants never use or stop using the e-health interventions [16]. Lack of engagement may arise mainly due to a lack of interest fueled by various other reasons that cause reduced user uptake and sustained interactions with a digital intervention [17]. High attrition marks dropout attrition, where many users do not return to fill in follow-up questionnaires since participants are not using, or are infrequently using, the intervention [16]. Thus, it is essential to design interventions that are appreciated and valued by the user to yield the optimal results of the ICT interventions.

Research highlights that a better understanding of the target users and their needs paves the way to design and develop interventions that respond to their needs. Thus, careful identification and analysis of user needs are essential [18]. In the current software development context, system features tend to be prioritised using limited volumes of qualitative user input [19]. This may lead to the design of undesirable feature sets for the end-users as the target audience may consist of a heterogeneous group of individuals that were not fully captured in the selected qualitative sample. The work of Lucy Yardley [20] emphasises the importance of combining qualitative and quantitative approaches to overcome the drawback of the single requirement elicitation technique. Thus, this current study aims to explore the findings of a previous quantitative analysis [21] carried out in the same study context to deepen the understanding of user needs and derive a concrete set of design requirements to feed into the design of digital occupational stress management intervention for software employees in Sri Lanka. Such a mixed methodological approach is deemed to provide the avenues to yield sufficient depth and breadth of user needs to feed the design of digital interventions.

## Methods

### Study context

The proposed study was conducted in Sri Lanka involving software employees working in software companies in the Colombo district. In the context of the Sri Lankan service sector, the IT-BPM (Information Technology and Business Process Management) industry marks the fourth-largest export revenue generator with a 130% value addition over the past decade [22]. This fastest-growing industry has over 600 IT-BPM companies in Sri Lanka, with more than 130,000 workers as of 2019 [23]. The current composition of the IT workforce statistics highlights that software engineering (39%) and software quality assurance (15%) amount to 54% of the job categories in the IT workforce. There is a gender imbalance where the Sri Lankan IT industry employs more males accounting for 66% of the total sector workforce. The industry has a young force where most employees belong to the age group of 25–35. The Sri Lankan IT industry is equipped with a high-skill workforce where 85% of the IT workforce has a bachelor’s degree or above qualification. More than 50% of the IT workforce is still in the early years of their careers [23]. Experience and education will jointly contribute to a mature and talented workforce. The recent statistics published marked that the highest level of attrition of the IT workforce was reported from IT companies at 9.7% compared to non-IT companies (5.5%). With the fast-paced industry and service environment, the competitive run to reach the next level, long working hours, and lack of work-life balance could contribute to workers’ stress-related health concerns [21, 24]. Thus, companies are taking numerous measures to safeguard their employees and provide a suitable work environment for their employees.

### Study design

This study is a sub-study within a larger project that aims further to explore the findings from the previous quantitative survey. The quantitative analysis [21] was conducted in the same context; it used an online questionnaire and collected 408 responses from Sri Lankan software employees. The questionnaire investigated the stressors, coping strategies and design preferences for ICT -supported stress management interventions. The study findings revealed that most software employees had a moderate stress level. They perceived work stressors and role stressors as common causes of stress. The most frequently used coping strategy was to seek social support, followed by digital activities, sports and physical exercises. The male and female design preferences varied significantly but only slightly based on their specific job categories. Moreover, findings suggested the necessity of further elicitation of user needs to support the design process.

Thus, this study involved an exploratory and qualitative approach to further explore the previous study’s findings to receive a definite set of user needs and design preferences of an ICT-supported intervention to manage occupational stress among Sri Lankan software employees. A multiple focus group approach was used as the data collection method. An online form (see Additional file 1) was created using the Stockholm University survey tool to recruit participants for the study. Several channels were utilised to disseminate the link to the software employees working in the Colombo-based software companies. These include publishing on social media platforms or groups for Sri Lankan software employees and direct emails to software companies in the Colombo district, which are listed in the SLASSCOM (Sri Lanka Association for Software Services Companies-the apex body of Sri Lankan software and services companies) members list. The participant recruitment form was available online for three weeks until 25 responses were collected. At the recruitment stage, consent to participation was collected online using the same condition. The recruited participants were purposefully added to the focus groups. The rationale for forming focus groups was based on the insights from the previous study [21] of 408 software employees who participated in the survey (see Additional file 2). The findings of this study [21] revealed gender-wise statistically significant differences in user preference for intervention features, support mechanisms and non-functional requirements. Males preferred physical exercises, conversations, and gaming components over females. The female group’s mean scores were high in support-seeking mechanisms. Moreover, females preferred to seek support from peers and professionals over their male counterparts. It is also noted that females had higher mean scores for non-functional requirements than males. Thus, gender was considered the primary attribute when establishing the focus groups, with one focus group dedicated to female participants. Next, the job category was crucial when allocating participants to each focus group. The employee age, years of experience and size of the company were not prioritised during group formation, but the distribution was considered to reflect the composition of the software industry. Accordingly, MW and ÅS created three focus groups (see Table 1).

**Table 1:** Demographics of focus group participants

Focus Group ID	Participant ID	Gender	Age Range	Job Category^a^	Years of Experience
Female Group (FG)	FG_1	Female	35–39	PM	9
	FG _2	Female	25–29	SE	4
	FG _3	Female	25–29	SE	2
	FG _4	Female	40–44	QA	15
	FG _5	Female	25–29	SUP	3
	FG _6	Female	25–29	QA	5
Male Group (MG)	MG _1	Male	25–29	SE	7
	MG _2	Male	35–39	QA	9
	MG _3	Male	40–44	PM	16
	MG _4	Male	30–34	SUP	6
	MG _5	Male	35–39	SE	11
	MG _6	Male	30–34	SE	7
	MG _7	Male	30–34	PM	8
	MG _8	Male	25–29	UI/UX	5
Mixed Group (XG)	XG_1	Female	30–34	BA	8
	XG _2	Female	25–29	QA	6
	XG _3	Male	35–39	SE	11
	XG _4	Male	35–39	SE	8
	XG _5	Male	30–34	QA	6
	XG _6	Female	25–29	QA	4
	XG _7	Male	25–29	UI/UX	4
	XG _8	Male	30–34	SE	9

^a^*SE* Software engineer, *QA* Quality assurance engineer, *UI/UX* User interface and user experience engineer, *PM* Project manager, *BA* Business analyst, *SUP* Implementation and support engineer

With the prevailing Covid-19 situation in the country at the time of the study (October 2021), the focus group discussions were conducted online via the Zoom platform. Based on the input received from the participant, convenient dates and times were decided. Initially, all the respondents were sent an email invitation specifying the chosen date and time for the discussion. Out of 25 participants, 23 confirmed their availability. Email reminders and the link to the meeting were sent three days and one day before the meeting, respectively. However, one previously registered participant did not join the session due to official commitments.

The meeting started 10 min before the scheduled time and was open for participants to join during the session. MW (female) played the moderator role in all three sessions. MW was the only research team representative for the online discussions. After each session, the research team had separate sessions and discussed the highlights of each session. MW is a senior lecturer in the profession and a PhD student in Computer and Systems Sciences. She has received quantitative and qualitative research training and has experience conducting studies in Sri Lanka involving in-depth interviews in both English and the native language, Sinhala. Before the commencement of the discussion, MW requested participant consent to make a digital recording of the session. Upon receiving approval, MW started the formal proceedings of the session by welcoming and introducing herself to the participants. Then an overview of the current study and the objectives of the study were presented using a PowerPoint presentation. During this presentation, the findings from the previous survey were introduced to create a background for the discussion. After the introduction, the discussion round started. During the meeting, participants were requested to reveal their thoughts and views on the presented ideas or express any new ideas. The discussion was conducted through a series of questions and sub-questions (see Additional file 3) to deepen the understanding of user needs and design requirements. All the discussions were carried out for nearly an hour and fifteen minutes. They were conducted in English or the participant’s native language (Sinhala), based on their preferences. Mainly, the conversations were carried out in English. However, it was visible that when participants elaborated on the experiences or jointly discussed some practical examples, they used Sinhala. To get the participants involved in the discussions, they were encouraged and posed with questions from time to time. MW used a field notebook to take notes during the focus group discussions (FGDs).

The number of FGDs was decided based on the commonly cited guideline saying that focus group research requires at least two groups for each defining demographic characteristic [25]. However, a comprehensive analysis of 40 focus group-based studies revealed that three focus groups were enough to identify the most prevalent themes within the data set [25]. Another essential aspect is the number of respondents invited for a discussion. Although it is generally accepted that between six and eight participants are sufficient, other studies have reported as few as four and as many as fifteen [26]. This was reconfirmed during the study as no new information was identified relevant to the study’s objectives at the point of three FGDs. At the end of the discussions, MW summarised the discussion points and sought confirmation from the participants. This information cross-validation among the participants helped to enhance the trustworthiness of the information.

### Data analysis

All the discussions were transcribed verbatim from digital recordings, and participant identification was removed. The Sinhala transcription was later translated by MW. The transcripts were manually analysed using the inductive thematic analysis [27] process and documented using Microsoft Excel. The inductive thematic analysis supports identifying, analysing and reporting patterns from responses or data, which allows the researcher to organise and describe the data in detail [27]. MW read and referred to the transcripts to familiarise herself with the content. The transcript sentences identified the meaning unit related to the study objective. Then, MW inductively coded the information in the transcript, discussing the codes with the second author (ÅS). Specified codes were classified into sub-themes/themes. Field notes were also reviewed during the coding process. Data was interpreted by analysing the code pattern among the participants. Emerged codes, sub-themes and themes were reviewed through peer debriefing by ÅS and another researcher (HS) who is an expert in occupational stress management. The consolidated criteria for reporting qualitative studies (COREQ)-32 items [28] were followed for reporting this study. The sample of the coding process and the COREQ-32 item checklist are presented in Additional file 4.

## Results

**Fig. 1:**
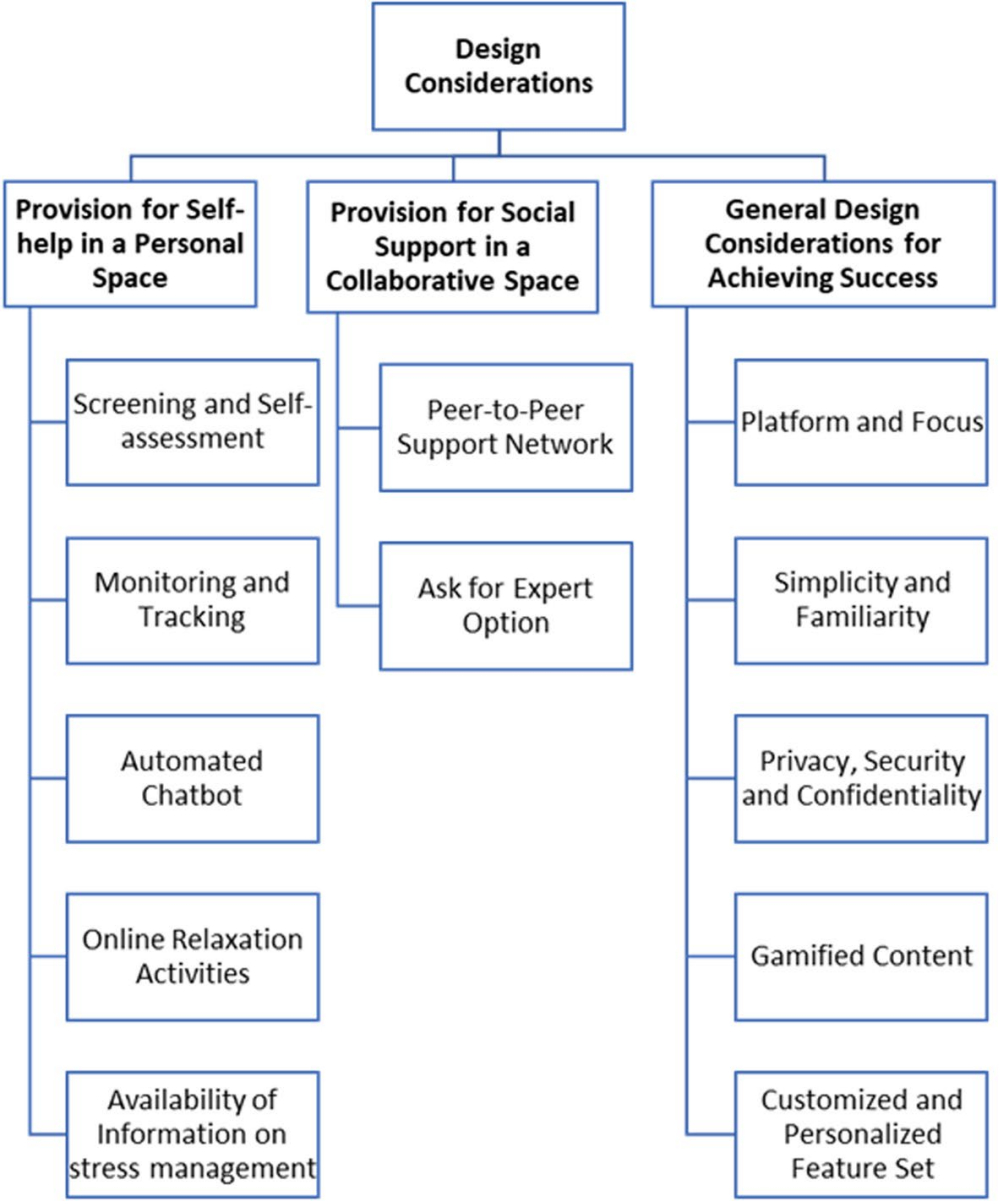
Design Considerations Arise from the Participants’ Perspective

As a result of the inductive thematic analysis, three major themes emerged. The themes and subthemes are represented in the coding tree below (see Fig. 1), and a sample of the coding process is attached in Additional file 5.

### Theme: Provision for self-help in a personal space

The first theme revealed the participants’ user preferences for features and activities they can engage in individually. These primarily focused on having features to assess, monitor stress and engage in simple activities that help to manage their stress levels. The first theme consists of five subthemes, as described below.

### Screening and self-assessment

Participants highlighted the importance of having a feature to screen stress levels online. They expressed their interest in using biomarkers to identify the stress levels connected with the available sensory features in their mobile devices. They also sought the possibility of having a simple questionnaire to answer and identify the current stress levels. They believed such a self-assessment feature would be useful to help them evaluate their present condition rather than seek external support.

*“If you have a platform that can identify your stress levels and stress factors and provide suitable activities based on preferences like reading a cooking recipe or sports or whatever is one thing (…).” [MG_3]*

*“An app can be integrated with our existing mobile devices and its sensors and readings to get to know whether the user is stressed or not.” [XG_1]*

*“If the app can facilitate identifying or recognise whether you are stressed, it is important. If the app automatically identifies that you’re stressed, maybe when a given threshold level is passed, the app notifies and responds to you accordingly.” [XG_4]*

### Monitoring and tracking

The participants’ preferences on monitoring and tracking possibilities spread across a wide range. They elaborated on their preference for background processes capturing user behaviours like eye strain, typing speed, social media and internet usage statistics, etc. They also sought the possibility of having a daily briefing and notification feature highlighting essential activities and upcoming deadlines. The participants expressed that they fall into stressful situations when they cannot manage their workload within the given timeframe. So, they believed this feature would help them plan their work and adjust their routines appropriately.

*“We get stressed because of poor time management, so it can have a feature to manage our time daily. I think we can reduce stress.” [FG_2]*

*“If the application can get information from what you are wearing or by analysing your calls or checking screen time, idle time, tracking what you are doing is important. If they are being captured and analysed automatically rather than we are constantly telling that this happened this time and this happened that time would be great.” [MG_4]*

*“If the app sends notifications based on our work plan deadlines and reminds us to start our work early or expedite the job, it would be great.” [XG_ 2]*

Moreover, Mixed Group participants highlighted the importance of a diary or journaling feature, giving them a personal space to write down their feelings and track their mood changes. In the Female Group, some participants shared their experiences regarding a similar application called “Happiness Calendar”, where employees could record their feelings from time to time. Then the application can summarise moods and display them using visual aids. They believed such a feature would help them recognise how their moods swing over time and quickly identify the root causes of stressful situations.

*“We are used to keeping a diary to write things that happened to us; and when something good or bad happens, we tend to write them there... so if the app also could support similar like a journaling feature is good. Then we can record and track them and keep them with us all the time.” [XG_3]*

*“The app can have a mood tracking feature so from time to time; users can record their current mood using a simple emoji so the mood tracking outcome may be displayed at the HR dashboards and individual dashboards well. Later these can be used for predictions even.” [XG_5]*

### Automated chatbot

Another feature requested by the participants is the automated chat option. They revealed their experience with similar applications like “Siri” and “Cortona” and sought the possibility of embedding a similar feature in the intervention. The participants elaborated that this feature could help them interact when bored or stressed. They also highlighted that chatbots could guide through required information on stress management and related activities. They also consider this type of conversation necessary when they cannot reveal matters openly to others but need someone to listen and provide emotional support.

*“I prefer less guidance from a third party. But automated help would be useful.” [MG_ 2]*

*“An app could minimise isolated situations and act as a companion. Like a chatbot.” [XG_7]*

### Online relaxation activities

The participants highlighted the importance of having simple artistic relaxation activities that they could perform online. Their preferences included the possibility of listening to relaxing music, playing their favourite playlist, mandala artwork, watching amusing videos, etc. They preferred content based on their preferences, while the intervention may suggest content based on their desired categories. Moreover, they emphasised adding a feature to offer physical activities that can be done within a limited time frame to make themselves more relaxed within a work setting. They believed this type of engagement could help them change their work pattern and relax while working.

*“As a feature, can we add fun activities that we can engage in simple, fun activities in less time?” [FG_3]*

*“(….) something to divert the thinking pattern from one angle to a different angle, maybe watching a movie or maybe doing some workouts, anything that is different from the usual pattern so that the trend will be broken (…).” [MG_5]*

*“Application must be simple and caters to our requirements. Many friends of mine listen to music while working, but I cannot work while listening to music. When I start working, I tend to work long hours without taking any breaks, so I find it difficult at the end of the day. So, if I can get a feature to remind me to take a break every hour, this would be a good feature.” [XG_6]*

### Availability of information on stress management

The participants emphasised the need for accurate information on stress and stress management activities. They also highlighted the possibility of recommending online books and content to read and refer to. They believed this informational support would help them find the right solution and motivate them to discover new ways of managing stressful situations.

*“(…) then the second part would have the knowledge to avoid stress or stress factors it may be training for eye exercises or simple stretching (..).” [MG_1]*

*“Receiving information and tips on stress management is also helpful. So, we can try to find a solution by ourselves.” [XG_8]*

Along with the user preference for individual activities, the participants also expressed their appreciation for having a collaborative environment with interactive activities that they can engage in to manage their stress levels. The next theme is dedicated to this purpose, revealing the user preferences for social support.

#### Theme: Provision for social support in a collaborative space

Similar to the first theme, the second emerged based on the participants’ feature preferences. These features focus on collaborative aspects where individuals expand their social circle to peers and experts to engage and seek support. The findings revealed two primary mechanisms; firstly, they sought the possibility of connecting with their peers, and secondly, they wanted to ask an expert for help.

### Peer-to-peer support network

Participants, especially the Female Group, underlined the importance of having a feature to share experiences and success stories, create discussion forums and have chat conversations among their colleagues. They also highlighted the importance of managing the visibility settings for their content. So, users could select a preferred audience and restrict the visibility of their posts and activities. They shared their experiences and explained that they have friend groups in the work environment where they share their concerns and problems and seek support. They believed that if this could be reflected in the virtual setting, they would be able to communicate more efficiently and seek help when necessary.

*“We have groups, at the moment also we have groups that we use to communicate with... these groups keep us updated, and whenever I get a problem though I cannot reveal it to everyone, I can expose it to my group members.” [FG_1]*

*“If we can have a collaborative platform, it is essential. This platform may support gathering as friends*, *team members or individuals with the same expertise; this will help share experience, generate creative ideas, collaborate, and support each other.” [XG_8]*

Some participants suggested having a peer community within similar job categories. They believed that people from similar jobs could share similar problems, and if they could work closely, they could find solutions more easily.

*“(….) that anyone can initiate a topic and the participants should be grouped based on each expert area, problems faced by the business analysts may not be the same problem set for the project managers. So if you group them in areas of expertise, you may realise that the same stress level was faced by some senior guy years ago; you may talk to them and will be able to act before it reaches the red light.” [MG_5]*

Participants highlighted that this community-based conversation feature would encourage them to engage in conversations actively and support others in their hard times.

### Ask for expert help

Apart from the peer support network, participants elaborated on their preference for a mechanism to seek support from experts in particular circumstances. They explained that it is necessary to seek professional help when the user is experiencing a severe mental health condition or needs extra support to manage the situation. Participants also highlighted that this communication should not be limited to the counsellors’ and stress experts’ support but also enable the possibility of conveying specific problems and concerns to the appropriate administrative levels with or without revealing user identity. They believed this expert involvement would help receiving professional support at their convenience. They also mentioned that when the employee’s conditions are notified to the employer, they will take remedial action to safeguard the employee by providing adequate resources and support. The participants believed this type of support within a work setting could place the employee in a safer place to combat work demands.

*“There can be severe cases as well. Where they may need counselling, and some may be on medical terms. So, if the system can alert or trigger such situations, an expert may jump in to provide necessary support without reaching the red line.” [MG_6]*

Apart from the features and functionalities of the intervention discussed in the first two themes, there were discussions on more general design preferences for the proposed intervention. Below, the final theme elaborates on such design preferences discussed at the FGDs.

#### Theme: General design considerations

The final theme is woven around the emerging points related to the non-functional requirements and factors that make users actively engage and continue using the proposed intervention. Outgoing from the functionalities and features of the intervention, the final theme discussed the design preferences uncovered by the participants. The user preference for the intervention platform, accommodating familiarity, simplicity, and maintaining privacy, security and confidentiality were discussed. Moreover, user preference for applying gamification and receiving customisable and personalisable content was also discussed. The five subthemes of this theme are presented below.

### Platform and focus

The focus groups responded to this aspect in similar ways. They preferred a hybrid solution rendered as both a web-based and a mobile-based application. The participants believed this would help them use the application conveniently anytime and anywhere using their own devices.

*“I prefer an online app that can be used on my phone and my laptop. Not a desktop or isolated application.” [FG_2]*

*“Currently, I’m using a mobile app, but I feel it’s better to support both mobile and web apps with a chrome plugin to capture our activities and screen time so we can get some sort of a guide to managing our time and work.” [XG_7]*

Moreover, Male Group and Mixed Group participants highlighted the importance of embedding wearable devices into the application. They thought it would help capture the required bodily measurements to identify the stress levels and capture the actual behaviours of the employee to customise the interventional content.

*“An app can integrate with our existing mobile devices and its sensors and readings to get to know whether the user is stressed.” [MG_4]*

Furthermore, focusing on the participants’ preference for the delivery mode of the application, they preferred a community-based intervention that supports collaborative features over a standalone application. All the focus groups generally raised this point, but the Female Group emphasised their high preference for community-based applications for communicating and engaging with peers.

*“Better to have a web application which looks like Facebook, but there are introverts and extroverts, so if it can have both options, it’s good. Because some don’t like to interact with the community so better to have the option to handle privacy based on user preferences.” [FG_3]*

*“It is good to have groups… so we can communicate our problems in the group and seek support.” [XG_2]*

### Simplicity and familiarity

The participants strongly emphasised the need for a simple and easy application without significant configurations. They also highlighted that the features should be user-friendly and easy to engage with. Some participants preferred to have features running in the background to capture user behavioural data automatically without disturbing their work. They also emphasised that the simple application and feature set would motivate users to use the system conveniently without significant effort.

*“My preference of the application would be like a lightweight background process which involves a one-time effort to configure and set up.”[MG_8]*

*“In my personal experience, I’m not using these health applications because they are complex to configure at the start. They can be simplified, so you don’t need much time to configure.” [MG_2]*

One crucial factor identified within the conversations is “familiarity”. The participants always tried to match their requirements with experience with similar applications. They highlighted Facebook when discussing the peer-to-peer network feature and its features supporting interactive conversations and engagements with peers. Moreover, they highlighted similar applications like Siri and Cortona to resemble their feature preferences when talking about the automated chat option. Further, during the discussion of the possibility of monitoring and tracking, participants highlighted a “Happiness Calendar” application and its features as their preferred mechanism to capture the mood. Moreover, when identifying suitable online relaxation activities, they emphasised the importance of having their usual activities on the applications, e.g. Mandala art, clay activities, etc. They believed such familiarity would help them to engage with the application efficiently.

*“Currently, I’m doing this Mandala Art when I feel stressed; I use this. I get a printout and try to colour. So, I think something similar would be good to have.” [FG_2]*

*“I’m used to having a clay ball with me. When I feel stressed, I try to make animals with it. So if I can see something like this, it would be great.” [FG_3]*

*“Our office has a dashboard where we see memories and past events pop up from time to time. So, it helps us refresh ourselves, and newcomers also get inspired. So some similar features may be added to the app.” [MG_6]*

### Privacy, security and confidentiality

The participants raised privacy, security and confidentiality as top priorities in the application. The participants were most concerned about the confidentiality of the data captured through the application and how such data could be managed securely. Moreover, they were also worried about securing privacy when engaging and communicating with unknown people outside their social circle. The contributors also sought the possibility of anonymous posting, guest user logins, hidden identity conversations with counsellors, etc. They emphasised the need for identity-revealing and masking options to support introverts and extroverts in carrying out their discussions and engagement at their own pace within a comfortable setting.

*“Can we keep both the types: registered and guest user access? Maybe some people do not like to introduce themselves.*” *[FG_5]*

*“A feature to reserve a slot with a counsellor, doctor or expert is also good. The company could also get notified based on such appointments. So, they are also aware of such a situation, but users should have the provision to nominate the levels to be notified. Also, there may be situations when users need to escalate something to HR without revealing their identity. So, both aspects should be covered.” [XG_5]*

### Gamified content

The participants embraced interactivity and engagement. They believed that the gamified content rendered in the application would intrinsically encourage users to engage with the application. They highlighted that users would be motivated to interact if the application rewarded their engagements through badges or stars. They believed members would be encouraged to follow the achievements of their peers when they are shared among peer groups. This would indirectly help them improve their well-being while assisting others. The participants also highlighted the importance of setting workout targets, e.g. step count, mind-calming activities, breathing exercises, etc. Then the application could remind them to achieve those targets during their busy work schedules. They thought this would help the user to break the usual work pattern and enjoy a quality break.

*“When members are contributing and supporting each other or practising health activities like walking, exercising, doing yoga, they may be recognised with a badge or star point system so individually they get motivated, and the other members also would get motivated.” [FG_4]*

*“It’s better to have different team challenges. I have one experience with a client where they provided a platform for us to do a step count challenge as a team; this encouraged us to do something out of work, even trying to walk while working in the office.” [XG_2]*

### Customised and personalised feature set

The participants preferred to have personalised content through the application customised according to their preferences and mental state conditions. Some participants emphasised their preference for online music activities, while others highlighted artistic activities. Participants had their preferred list of activities they would like to perform in stressful situations. Similarly, some participants wanted to log in with their actual identity, whereas others wanted a virtual login. Some participants expected recurring notifications, whereas some thought it disturbed their work. Collectively, participants appreciated tailor-made content based on their preferences. They thought such a personalised set of features would make the application more usable and motivate users to engage more.

*“I think it is better to have the option to set security levels and the possibility to add group members based on my preference*.” *[FG_1]*

*“Application must be simple and cater to our requirements. Many friends of mine listen to music while working, but I cannot work while listening to music.” [MG_3]*

*“The app should have activities catering to both levels. I would say one is individual-level activities and then the group level where we can have chat services and conversations to discuss our things.” [XG_5]*

The final theme elaborated on the design considerations to achieve success. As evident in the literature, most digital health intervention is subjected to less user engagement and adherence. Thus, this theme elaborated on the design considerations that emerged in the participants’ discussion to mitigate such engagement and adherence issues in the proposed intervention. Such considerations evolved on the platform preferences, the importance of embedding gamified elements, and keeping the intervention personalised while assuring simplicity.

## Discussion

The current study used a qualitative approach to further explore and refine user needs and identify requirements for designing a digital stress management intervention for software employees in Sri Lanka. The focus group discussions facilitated in-depth conversations with the participants to deepen the understanding of the user needs and to co-generate some design ideas to be incorporated into the intervention. The findings are three-folded. Firstly, it revealed user preference for personal space with different stress management activities. Secondly, it exposed the user preference for collaborative space with multiple support mechanisms. Finally, it elaborated on the user preferences on various aspects like focus, platform, privacy and confidentiality, content customizability, etc.

The previous survey [21] showed user preference for individual activities and collaborative platforms. This was further elaborated in the FGDs, and the discussions also revealed user preference for having two separate spaces in a single intervention. On the one hand, they focused on a personal space with self-help options. They wanted to engage in personalised activities to identify and manage their stress levels with automated or minimal guidance. This activity list may be generated based on their familiarity and interest while receiving the relevant expert recommendations. On the other hand, the participants were interested in sharing their ideas and seeking support from their social circles. They were keen on learning and engaging with peers to enhance their skills and get positive motivation to carry out healthy behaviours. Mainly female participants elaborated on their preference for peer-supported mechanisms. The previous study [21] found that support-seeking behaviour from peers was most evident in Female employees, which was also confirmed in the FGDs. Not only that, but participants also appreciated the expert support and believed such help would guide them in the right direction, especially when the stress symptoms are severe. A systematic review [14] of digital occupational stress interventions shows that most interventions focus on individuals, and they primarily focus on providing knowledge and skills to cope with stress effectively [29]. However, the major challenge identified in such interventions is less engagement and high attrition [30]. Previous studies show that peer support mechanisms and expert support could increase engagement and adherence to the intervention [31, 32]. Thus, personal space with a collaborative space allows the user to engage in a wide range of interventional activities, may improve the efficiency of the intervention and address the generic challenge of poor adherence, engagement and attrition associated with digital interventions [32, 33].

During the previous quantitative study [21], most respondents preferred to use a mobile-based application followed by a web-based one. This idea was further confirmed and deepened by the focus group participants. They highlighted their interest in a cross-platform application accessible via mobiles and the web. Additionally, they sought the possibility of embedding sensors in their existing mobile devices in the intervention. Their selection was primarily based on their familiarity, availability and accessibility of the devices and technologies. The current spectrum of intervention delivery modalities includes text messages, smartphones, smart devices, computers and other handheld devices [10, 22, 23]. The selection of the modalities will restrict the capabilities of technology embedded in the intervention. For instance, selecting devices with larger screens allows the delivery of a high volume of information. In contrast, choosing small devices like smartphones and smartwatches would restrict information on a single screen.

The next factor that emerged was related to adding automation and gamified content into the intervention. The participants highlighted their interest in receiving automated content based on sensory data or user behaviours. However, the previous quantitative study [21] yielded low rates for sensory-based applications, which a few participants discussed in the open alternatives option. But the FGDs open discussions on the applicability of sensory technologies within the intervention. Participants believed that if the application automatically recognises increased stress levels or work routines and responds according to the condition, it will help manage their activities. This type of passive data collection mechanism facilitates receiving insights into user behaviours without disturbing the usual work routines of an individual [34]. Multiple studies have shown the possibility of using sensors in mobile devices for real-time stress detection and monitoring purposes [10]. They also look forward to having an automated chatbot system to keep them engaged with the intervention. They thought such agents would support and guide them through hard times and act as companions. Previous studies show that such notifications, guidance and support improve user engagement with the intervention [14, 35].

The other essential aspect captured in the discussions was how the gaming concept could be embedded in the intervention. The previous quantitative study [21] showed less mean score value for the user preference for gaming modules. But during the FGDs, the participants elaborated on their importance for engaging in physical gaming elements like group-level activities to improve their health behaviours. They shared experiences of different group challenges they had been involved in, which helped them achieve the set targets as a team. During the previous quantitative study [21], participants preferred group activities, which was reconfirmed and elaborated further during the current study. These FGDs clarified and deepened the understanding of what activities they would like to engage in. They believed such activities helped them increase social connectedness while cultivating positive, healthy behaviours among individuals. Moreover, the participants were interested in having mini-games or activities they could play individually for short periods. One new point that emerged during the discussion was the applicability of the gamification element within the intervention. This point was not revealed in the previous quantitative study [21]. They highlighted the importance of acknowledging their commitment to positive behavioural changes through incentives like badges, stars, etc. They believed such recognition would motivate them to engage more with the intervention. There is considerable research evidence that serious games positively contribute to individuals’ mental health conditions [36, 37]. Though its primary focus is not to deliver entertainment, such gaming elements help effectively leverage individuals’ knowledge, skills, and attitudes toward mental health conditions [38]. Moreover, research has shown that digital game interventions provide a unique and interactive platform for its players to facilitate learning among players and improve the skill set of individuals [39]. Assessing the current technological support available for games and adding gaming elements to the intervention may enhance the range of interventions while supporting prevention, intervention and treatment [36].

The lack of personalisation is a significant drawback in digital interventions causing a lack of user engagement [15]. This fact was hardly discovered during the previous quantitative study [21]. Only a few participants discussed this matter at the open alternative option. However, survey participants showed their preference for tailor-made content facilities. The focus group participants showed interest in an intervention matched with their context, experience and values rather than a one-size-fits-all solution. They were keen on the perceived fit of the intervention and sought the possibility of customising the content based on their preferences, interest, and familiarity. They identified a positive correlation between the perceived fit and the perceived usefulness. Perceived usefulness refers to the users’ experience with the intervention and their perceptions of whether it would be helpful to them [31]. The participants believed that if the intervention can cater to the individual need, it will increase its usefulness. Following the previous survey results [21], focus group participants elaborated on their preference not only with the content of the intervention but also expected to see similar tailor-made facilities in the privacy and security levels, guidance and support. Literature shows that the increased perceived usefulness would encourage and motivate users to engage and adhere to the intervention [31]. Thus, it is vital to recognise the individual needs before designing the intervention.

This study has some limitations, which would threaten the potential benefits and validity. As the study is based on self-reported data, the reporting could be influenced by the group setting and stress among the participants. Nevertheless, they reported data in their own words during the investigation. The collected data could be estimated in any way other than by self-reports, and the conclusions should be drawn carefully. Given that the study involved a sample that represents the gender, age, and job categories of the target population, the study findings can be related to the population, ensuring the external validity of the study. Since the global software industry’s work culture resembles the Sri Lankan software industry, the findings may also be relevant for a broader geographical area. The qualitative approach adopted during the study provided the opportunity to engage in rich conversation with the target users and gain an in-depth understanding of their user needs. Yardley’s [40] work on the ‘Person-Based’ approach also illustrates the importance of involving qualitative research to understand and respond to the perspectives of potential users at the early stage of the design development of an intervention. It is believed that deriving perspectives from previous literature and quantitative studies could provide insights on intervention components that are likely to be effective or cost-effective but often do not provide guidance on what components are important or how they should be implemented in a particular context [40]. Thus, this application of qualitative explorations of the user needs on the findings of previous quantitative studies has provided a balanced approach to elicit a concrete set of user needs to inform the intervention design in the next stage. These findings could provide insights for designers to design ICT-supported stress management interventions in the next cycle.

## Conclusion

Digital health interventions are considered a promising solution in the contemporary technological and societal context. However, considerable research evidence on constraints embedded in such interventions hinders the potential benefits of the intervention. The current study could be part of a process that identifies user needs to design a digital intervention for occupational stress management for software employees in Sri Lanka. The insights received from the FGDs confirmed and deepened the understanding of the findings of the previous quantitative study. The results confirmed the importance of incorporating personal and collaborative space within a single intervention. Such personal space may carry several functional modules that could be selected based on the user’s preference. Discussions on the collaborative space further revealed the user preference for guidance and support mechanisms. The users preferred to receive peer support followed by support and guidance from a professional. The findings of this study also revealed the importance of adding gamified elements to the intervention. Further, it showed the importance of embedding physical gaming elements, which can be performed as group challenges. Moreover, in-depth discussions were taken on the importance of personalising the intervention. The participants highlighted the importance of tailor-made content and other non-functional requirements like security options, visual elements, etc. In a future study, the yielded concrete set of feature requirements and design preferences will incorporate into the designs of ICT-supported interventions for occupational stress management among software employees in Sri Lanka.

## Supplementary Information

**Additional file 1.** Participant recruitment form.

**Additional file 2.** Survey used to collect data from software employees.

**Additional file 3.** Interview Guide used during the FGDs.

**Additional file 4.** COREQ 32-item checklist.

**Additional file 5.** The sample of the coding process.

## Data Availability

Data gathered or analysed during this study are included in this published article at an aggregate level. The original transcripts of the focus group discussions used in the thematic analysis process are available on reasonable request from the corresponding author.

## Abbreviations

BA: Business analyst
FGDs: Focus group discussions
GCS: Global consumer study
ICT: Information and communication technology
IT-BPM: Information technology and business process management
PM: Project manager
SE: Software engineer
SLASSCOM: Sri Lanka association of software and services company
SMIs: Stress management interventions
SUP: Implementation and support engineer
QA: Quality assurance engineer
UI/UX: User interface and user experience engineer
