# Supplementary material for: Exploration of user needs and design requirements of a digital stress management intervention for software employees in Sri Lanka: a qualitative study
Source: BMC Public Health. 2023 Mar 27;23:566. doi: 10.1186/s12889-023-15480-7 (PMC10041489; doi:10.1186/s12889-023-15480-7)
Supplement: Supplementary file 1 — Additional file 1. Participant recruitment form. [file 12889_2023_15480_MOESM1_ESM.pdf]

This online study is a part of a PhD project by Manoja Weerasekara, at the Department of Computer and System Sciences at Stockholm University, Sweden.

The purpose of this study is to **recruit participants for a focus group discussion** (online) to investigate current stress management practices use by software employees and to understand their perception of ICT supported occupational stress management interventions. The study only focuses on the stress associated in the working environment. **As an employee of the software industry** we kindly request your participation to support the requirement gathering process.

This survey will take only 2-3min to complete.

We are very appreciative of the time you take to assist in our study. If you have any queries related to this survey, please contact me through manoja@nsbm.lk or manoja@dsv.su.se

1. Name (to appear during the discussion time)

2. I'm a

- ○ Male
- ○ Female

3. I belong to age category of

- ○ 18-24
- ○ 25-34
- ○ 35 and above

4. What is your marital status?

- ○ Married
- ○ Single, Never Married
- ○ Single, Divorced or Widowed

5. Highest Education Qualification that you have

- ○ Up to Higher Diploma Level
- ○ Bachelor
- ○ Postgraduate

6. Job Category

- ◯ Software Engineer (SE)
- ◯ Quality Assurance Engineer (QA)
- ◯ User Experience Engineer (UI / UX)
- ◯ Project Manager (PM)
- ◯ Business Analyst (BA)
- ◯ Implementation and Support Engineer (SUP)
- ◯ Database Administrator (DBA)
- ◯ Other, please specify

other,

7. Employment Type

- ◯ Permanent
- ◯ Contract

8. Years of Work Experience

- ◯ 0-3
- ◯ 4-8
- ◯ 9 and above

9. Size of the Company

- ◯ Small (1-49 employees)
- ◯ Medium (50-99 employees)
- ◯ Large (100 employees and above)

10. Preferred Discussion Time

	Morning (any time between 9-12pm)	Afternoon (anytime between 1-4pm)	Evening (after 6pm)
Wednesday	○	○	○
Friday	○	○	○
Saturday	○	○	○
Sunday	○	○	○

Please specify your preferred time

11. Preferred Language for the Discussion

- ○ Sinhala
- ○ English
- ○ Mix (English and Sinhala)

12. How can we reach you Mobile No:

13. How can we reach you Email:
