# Supplementary material for: Exploration of user needs and design requirements of a digital stress management intervention for software employees in Sri Lanka: a qualitative study
Source: BMC Public Health. 2023 Mar 27;23:566. doi: 10.1186/s12889-023-15480-7 (PMC10041489; doi:10.1186/s12889-023-15480-7)
Supplement: Supplementary file 2 — Additional file 2. Survey used to collect data from software employees. [file 12889_2023_15480_MOESM2_ESM.pdf]

Stockholm University

The purpose of this study is to investigate current stress management practices use by software employees and to understand their perception of ICT supported occupational stress management interventions. The study only focus on the stress associated in the working envioronment.

As an employee of the software industry we kindly request your honest information, and thoughtful suggestions to make this objective a sucess.

This survey will take only 5-8min to complete.

We are very appreciative of the time you take to assist in our study. Thank You So Much...!!

1. I'm a

- ○ Male
- ○ Female

2. I belong to age category of

- ○ 18-24 Years
- ○ 25-34 Years
- ○ 35- 44 Years
- ○ 45-54 Years
- ○ 55-64 Years
- ○ 65 and over

3. What is your marital status?

- ○ Married
- ○ Single, Never Married
- ○ Single, Divorced
- ○ Single, Widowed

4. If you have children living at home, please specify the no. in respective age categories.

No Children	
Less than 4 years old	
4 to 12 years old	
13 to 18 years old	
19 and over	

5. Highest Education Qualification that you have

- ○ GCE A/L or Less
- ○ Certificate/Diploma/ Higher Diploma level
- ○ Bachelor’s Degree
- ○ Postgraduate qualification (Postgraduate Diploma, Masters, PhD,etc.)

6. My job title?

Other

7. I have..... years of experience in the software industry

8. My employment is on

- ◯ Contract Basis
- ◯ Permanent Basis

9. Size of the company

- ◯ Small (Less than 49 employees)
- ◯ Medium (50-99 employees)
- ◯ Large (More than 100 employees)

10. Do you think that your family-work life is well balanced?

- ◯ Yes
- ◯ To a certain extent
- ◯ No
- ◯ Not sure

11. What is your habitual sleep like?

- ◯ Very Calm
- ◯ Fairly Calm
- ◯ Neither Calm nor Restless
- ◯ Quite Restless
- ◯ Very Restless

12. How would you rate your average stress level at work 1-No Stress and 10- Highest

☆☆☆☆☆☆☆☆☆☆

13. What makes you stressed at your work (Select Where Applicable)

	Strongly Agree	Agree	Neutral	Disagree	Strongly Disagree
Long Working Hours	○	○	○	○	○
Unmanageable Workload	○	○	○	○	○
Tight deadlines and Poor Deadline Management	○	○	○	○	○
Poor Relationships with Colleagues	○	○	○	○	○
Rapidly Changing Nature of Technology & Requirement (Stay up-to date with technology)	○	○	○	○	○
Unsupportive Boss or Organizational Culture	○	○	○	○	○
Few Career Growth Opportunities	○	○	○	○	○
Assuming Different Roles in the Same Project or in Different Projects (Disturbances)	○	○	○	○	○
Lack of Technical Expertise	○	○	○	○	○
Lack of Support from Family or Family Commitments	○	○	○	○	○
Presure being a team manager/leader and/or managing subordinates	○	○	○	○	○
Gray Areas on development requirements	○	○	○	○	○
Client Interaction/ Pressure Arise from the Clients (from both internal and external parties)	○	○	○	○	○

	Strongly Agree	Agree	Neutral	Disagree	Strongly Disagree
Poor Project Management	○	○	○	○	○
Insufficient Monetary/ Non-monetary Incentives/ Transport and Other Benefits	○	○	○	○	○
Organizational Setting and Work Environment	○	○	○	○	○
Insifficient Leave Facility (Casual, Medical, Annual, Parental Levales, etc)	○	○	○	○	○
Problem of handling multiple responsibilities (In work as well as in the family)	○	○	○	○	○

Other

14. In case of stressful situation what are the activities you involve in to reduce your stress levels?

	Rarely (Never)	Occasionally	Sometimes	Fairly Often	Very Often (Always)
Listen to music	○	○	○	○	○
Watch videos or movies	○	○	○	○	○
Engage in religious activities	○	○	○	○	○
Practice Yoga	○	○	○	○	○
Do meditation	○	○	○	○	○
Walk	○	○	○	○	○
Practice Deep breathing	○	○	○	○	○
Practice Muscle relaxation activities	○	○	○	○	○
Do Singing and/or Dancing	○	○	○	○	○
Do Painting	○	○	○	○	○
Hanging around with friends and family (Go on trips, partying, chit-chat, etc.)	○	○	○	○	○
Play digital games (computer games, online games, mobile games, etc.)	○	○	○	○	○
Use social media (Use FB, WhatsUp, Tweeter,etc)	○	○	○	○	○
Engage in Physical Activities/Sports (e.g. Badminton, Cricket, Swimming)	○	○	○	○	○
Smoking/Consume Alcohol	○	○	○	○	○

Other

15. When your job becomes a source of conflict and tension leading to stress, you will

	Rarely (Never)	Occasionally	Sometimes	Fairly Often	Very Often (Always)
Talk to my supervisor/boss and request support	○	○	○	○	○
Talk to HR and request support	○	○	○	○	○
Talk to co-workers and request support	○	○	○	○	○
Talk to friends and family members and request for support	○	○	○	○	○
Try to solve it by your own	○	○	○	○	○

16. How do you usually perform activities to reduce your stress levels? (select all applicable)

	Rarely (Never)	Occasionally	Sometimes	Fairly Often	Very Often (Always)
Individually/Alone	○	○	○	○	○
With a close friend or Partner	○	○	○	○	○
In a group	○	○	○	○	○
As an online activity	○	○	○	○	○
As an offline (Physical Activities)	○	○	○	○	○

Comment

17. Do you know about any ICT tools available for stress management? (E.g. Websites/mobile apps/Online forums/etc.)

- ◯ Yes
- ◯ No

18. Have you ever used such applications?

- ◯ Yes
- ◯ No

19. Are you interested in using ICT supported tools to manage your stress levels?

- ◯ Yes
- ◯ No
- ◯ Not Sure

20. If you are interested to use ICT supported tools to manage your stress level, what is your preferred platform?

	To a Great Extent	Somewhat	Very Little	Not at all
Mobile applications	◯	◯	◯	◯
Web application	◯	◯	◯	◯
Wearable sensors	◯	◯	◯	◯
Standalone application (Desktop applications)	◯	◯	◯	◯
Hybrid (combination of above options)	◯	◯	◯	◯

21. What features would you like to see on such platform? (select all applicable)

- ☐ Audio and Video clips
- ☐ Gaming components
- ☐ Motivational and feedback notifications
- ☐ Physical Exercises
- ☐ Mental relaxation activities
- ☐ Text or voice-based conversations
- ☐ Chat Bot
- ☐ Online Communication Platform/ Social Networking Platform to connect with people **inside** your organization
- ☐ Online Communication Platform/ Social Networking Platform to connect with people **outside** your organization
- ☐ Other

Please specify

22. What type of online support that you prefer in ICT supported tool to manage stress [Support and interaction in the form of: Messages, feedback, conversations, guidance, etc.,]

	Very Important	Important	Moderately Important	Slightly Important	Not Important
Support from a counsellor	○	○	○	○	○
Support from Peers/Colleagues/Friends	○	○	○	○	○
Computer based Guidance	○	○	○	○	○
Self-help or manage by myself option	○	○	○	○	○

23. What are the other features that you find important in ICT applications for stress management?

	Very Important	Important	Moderately Important	Slightly Important	Not Important
Confidentiality	○	○	○	○	○
Security	○	○	○	○	○
Content Accuracy	○	○	○	○	○
Customizable/tailor made content	○	○	○	○	○
Interactive components	○	○	○	○	○

Other,Please specify

24. Please specify other features and functions in ICT supported tools that might help you to manage high stress level & motivate you to use such system
