# Supplementary material for: Exploration of user needs and design requirements of a digital stress management intervention for software employees in Sri Lanka: a qualitative study
Source: BMC Public Health. 2023 Mar 27;23:566. doi: 10.1186/s12889-023-15480-7 (PMC10041489; doi:10.1186/s12889-023-15480-7)
Supplement: Supplementary file 3 — Additional file 3. Interview Guide used during the FGDs. [file 12889_2023_15480_MOESM3_ESM.pdf]

Discussion Guide:

Introduction

Good evening and welcome to our session.

Thanks for taking the time to join us to support our requirement gathering phase of ICT supported stress management interventions. I'm Manoja Weerasekara from Stockholm University. This is part of my PhD study where we focus on designing and developing an ICT intervention for occupational stress management. In the next phase, I will do a small presentation to describe the study's aim and objectives.

There are no wrong answers but rather different points of view. Please feel free to share your point of view, even if it differs from what others have said. Keep in mind that we're just as interested in negative comments as positive comments, and at times the negative comments are the most helpful.

We're recording the session because we don't want to miss any of your comments. We will use your first name or the given name during the session, but they will not appear in our reports. You may be assured of complete confidentiality.

Question Guide

1. How would you usually manage that when your job becomes a source of conflict and tension leading to stress?

2. If we are to design a technological intervention to manage stress
   2.1 What sort of a platform do you most prefer? (Mobile app, web app or online community platform) <moderator would provide a small description of each option)
   2.2 What sort of features would you like to see?
   2.3 What sort of non-functional requirements would you like to see?
   2.4 What sort of support and guidance you would like to see (fully guided, minimal guided, no guidance-self-help/ support from peers, support from counsellors,etc.)

3. Research shows that though digital interventions are promising, they are subjected to various constraints like lack of user motivation, lack of engagement, high attrition, etc. So what will make you motivated to use such interventions or applications?

Conclusion:

1. Summarize with confirmation,
2. Review the purpose and ask if anything has been missed,
3. Thanks and dismissal
