# Supplementary material for: Exploration of user needs and design requirements of a digital stress management intervention for software employees in Sri Lanka: a qualitative study
Source: BMC Public Health. 2023 Mar 27;23:566. doi: 10.1186/s12889-023-15480-7 (PMC10041489; doi:10.1186/s12889-023-15480-7)
Supplement: Supplementary file 4 — Additional file 4. COREQ 32-item checklist. [file 12889_2023_15480_MOESM4_ESM.pdf]

Additional file 3. Consolidated criteria for reporting qualitative studies (COREQ): a 32-item checklist

Developed from:
Tong A, Sainsbury P, Craig J. Consolidated criteria for reporting qualitative research (COREQ): a 32-item checklist for interviews and focus groups. *International Journal for Quality in Health Care*. 2007. Volume 19, Number 6: pp. 349 – 357

Item	Guide questions/description	Reported on Page #	Line #
**Domain 1: Research team and reflexivity**			
Personal Characteristics			
1. Inter viewer/facilitator	Which author/s conducted the interview or focus group?	4	99
2. Credentials	What were the researcher's credentials? E.g. PhD, MD	4	100
3. Occupation	What was their occupation at the time of the study?	4	99
4. Gender	Was the researcher male or female?	4	99
5. Experience and training	What experience or training did the researcher have?	4	100-101
Relationship with participants			
6. Relationship established	Was a relationship established prior to study commencement?	3	93
7. Participant knowledge of the interviewer	What did the participants know about the researcher? e.g. personal goals, reasons for doing the research	4	103-104
8. Interviewer characteristics	What characteristics were reported about the interviewer/facilitator? e.g. Bias, assumptions, reasons and interests in the research topic	4	104-105
**Domain 2: study design**			
Theoretical framework			
9. Methodological orientation and Theory	What methodological orientation was stated to underpin the study? e.g. grounded theory, discourse analysis, ethnography, phenomenology, content analysis	2<br><br>4	76<br><br>124-126
Participant selection			
10. Sampling	How were participants selected? e.g. purposive, convenience, consecutive, snowball	3	77-80
11. Method of approach	How were participants approached? e.g. face-to-face, telephone, mail, email	2,3	77-80
12. Sample size	How many participants were in the study?	3	81
13. Non-participation	How many people refused to participate	4	95-97

	or dropped out? Reasons?		
Setting			
14. The setting of data collection	Where was the data collected? e.g. home, clinic, workplace	3	92-97
15. Presence of non-participants	Was anyone else present besides the participants and researchers?	3	98-100
16. Description of sample	What are the important characteristics of the sample? e.g. demographic data, date	3	Table 1
Data collection			
17. Interview guide	Were questions, prompts, guides provided by the authors? Was it pilot-tested?	4	108-111 Additional file 3
18. Repeat interviews	Were repeat interviews carried out? If yes, how many?	4	110-120
19. Audio/visual recording	Did the research use audio or visual recording to collect the data?	4	102-103
20. Field notes	Were field notes made during and/or after the interview or focus group?	4	112
21. Duration	What was the duration of the interviews or focus group?	4	109-110
22. Data saturation	Was data saturation discussed?	4	113-120
23. Transcripts returned	Were transcripts returned to participants for comment and/or correction?	4	120-121
**Domain 3: analysis and findings**			
Data analysis			
24. Number of data coders	How many data coders coded the data?	4	128-129
25. Description of the coding tree	Did authors provide a description of the coding tree?	Figure 1	Additional File 5
26. Derivation of themes	Were themes identified in advance or derived from the data?	4	129-130
27. Software	What software, if applicable, was used to manage the data?	4	124-125
28. Participant checking	Did participants provide feedback on the findings?	4	120-121
Reporting			
29. Quotations presented	Were participant quotations presented to illustrate the themes/findings? Was each quotation identified? e.g. participant number	5-10	152-366
30. Data and findings consistent	Was there consistency between the data presented and the findings?	5-12	NA
31. Clarity of major themes	Were major themes clearly presented in the findings?	5-12	NA
32. Clarity of minor themes	Is there a description of diverse cases or discussion of minor themes?	5-12	NA
