# Supplementary material for: Exploration of user needs and design requirements of a digital stress management intervention for software employees in Sri Lanka: a qualitative study
Source: BMC Public Health. 2023 Mar 27;23:566. doi: 10.1186/s12889-023-15480-7 (PMC10041489; doi:10.1186/s12889-023-15480-7)
Supplement: Supplementary file 5 — Additional file 5. The sample of the coding process. [file 12889_2023_15480_MOESM5_ESM.pdf]

Additional file 2. Sample coding process

Meaning Unit	Codes	Subthemes	Themes
: "if you have a platform that can identify your stress levels and stress factors and provide suitable activities based on preferences like reading a cooking recipe or sports or whatever is one thing (...)."	Identify stress levels	Screening and Self-assessment	*Provision for Self-Help: Personal Space*
"If the app sends notifications based on our work plan deadlines and reminds us to start our work early or expedite the job, it would be great."	Monitor<br>Reminders	Monitoring and Tracking	
"App could minimise the isolated situation and act as a companion. Like a chatbot."	Chatbot	Automated Chatbot	
"as a feature, can we add fun activities that we can engage in simple, fun activities in less time?"	Simple activities	Online Relaxation Activities	
"Receiving information and tips on stress management is also helpful. So we can try to find a solution by ourselves."	Information	Availability of Information on stress management	
"We have groups, at the moment also we have groups that we use to communicate with.. these groups keep us updated, and whenever I get a problem though I cannot reveal it to everyone, I can expose it to my group members."	Community<br>Peer groups<br>Support	Peer-to-Peer Support Network	*Provision for Social Support: Collaborative Space*
"If the app has a feature to reserve a slot with a counsellor or doctor or expert, that would also be good (....)."	Counsellors<br>Support	Ask for Expert Option	
"Better to have a web application which looks like Facebook, but there are introverts and extroverts, so if it can have both options, then it's good.	Web<br>Facebook	Platform and Focus	*General Design Considerations for Achieving*
"In my personal experience, I'm not using these health applications because they are complex to configure at the start. They can be simplified, so you don't need much time to configure."	Simple<br>Automated	Simplicity and Familiarity	

"(....) there may be situations users need to escalate something to HR without revealing their identity. So, both the aspects should be covered."	Confidential	Privacy, Security and Confidentiality	*Success*
"It's better to have different team challenges. I have one experience with a client where they provided a platform for us to do a step count challenge as a team; this encouraged us to do something out of work even some tried to walk while there are working in the office."	Challenges<br><br>Game elements	Gamified Content	
" The app should have activities catered for both levels. I would say one is individual-level activities and then the group level where we can have chat services and conversations to discuss our things."	User preference<br><br>Capacity to select and change	Customized and Personalized Feature Set	
